# The role of extracellular vesicles when innate meets adaptive

**DOI:** 10.1007/s00281-018-0681-1

**Published:** 2018-04-03

**Authors:** Tom Groot Kormelink, Sanne Mol, Esther C. de Jong, Marca H. M. Wauben

**Affiliations:** 1 Department of Experimental Immunology, Academic Medical Center, University of Amsterdam, Amsterdam, The Netherlands; 2 Department of Biochemistry and Cell Biology, Faculty of Veterinary Medicine, Utrecht University, Utrecht, The Netherlands

**Keywords:** Extracellular vesicle, Exosome, Microvesicle, Microparticle, Innate immune cells, Adaptive immunity

## Abstract

Innate immune cells are recognized for their rapid and critical contribution to the body’s first line of defense against invading pathogens and harmful agents. These actions can be further amplified by specific adaptive immune responses adapted to the activating stimulus. Recently, the awareness has grown that virtually all innate immune cells, i.e., mast cells, neutrophils, macrophages, eosinophils, basophils, and NK cells, are able to communicate with dendritic cells (DCs) and/or T and B cells, and thereby significantly contribute to the orchestration of adaptive immune responses. The means of communication that are thus far primarily associated with this function are cell-cell contacts and the release of a broad range of soluble mediators. Moreover, the possible contribution of innate immune cell-derived extracellular vesicles (EVs) to the modulation of adaptive immunity will be outlined in this review. EVs are submicron particles composed of a lipid bilayer, proteins, and nucleic acids released by cells in a regulated fashion. EVs are involved in intercellular communication between multiple cell types, including those of the immune system. A good understanding of the mechanisms by which innate immune cell-derived EVs influence adaptive immune responses, or vice versa, may reveal novel insights in the regulation of the immune system and can open up new possibilities for EVs (or their components) in controlling immune responses, either as a therapy, target, or as an adjuvant in future immune modulating treatments.

## Introduction

For a long time, it has been a general principle in immunology that the development of effector T cells (Th1, Th2, Th17, or regulatory T cells) is directly driven by antigen-primed dendritic cells (DCs) [1]. However, it is now more and more recognized that both at inflammatory sites and in secondary lymphoid structures, DCs can interact with innate immune cells (i.e., mast cells, neutrophils, macrophages, eosinophils, basophils, and natural killer (NK) cells) resulting in the modulation of DC migration and function. These interactions subsequently influence T cell proliferation and/or polarization into effector cell subsets [2–9]. Moreover, the concept that innate immune cells can directly communicate with CD4^+^ and CD8^+^ effector T cells, regulatory T cells, γδ-T cells, and B cells, and thereby influence each other’s function, adds another layer of complexity in the regulation of innate and adaptive immune responses [10–16]. These accumulating new insights indicate that in contrast with the conventional idea that innate immune cells only participate in immune responses as terminal effector cells, they also significantly contribute to the initiation and shaping of adaptive immune responses.

Mechanisms that are involved in the cross-talk between innate and adaptive immunity obviously include cell-cell contacts and the release of a diverse array of soluble factors, including cytokines and chemokines. Another increasingly investigated and appreciated mechanism via which cells may exert their modulatory effect is through the release of extracellular vesicles (EVs). EVs are submicron structures, typically of 50 to 200 nm in size, and released by cells in a regulated fashion [17]. EVs can either be formed and released by outward budding from the plasma membrane (then often termed microparticles or microvesicles) or formed as intraluminal vesicles in endosomal multivesicular bodies and released (as the so-called exosomes) from cells upon fusion of the multivesicular body with the plasma membrane [17, 18]. Since the route of biogenesis cannot be determined once the different vesicle subsets are found in biological fluids or culture supernatants due to a lack of discriminatory markers, they are collectively termed EVs [19]. The content of EVs entails lipids, proteins, and nucleic acids, and therefore, EVs can be seen as multi-component communication devices. The selective incorporation of this cargo depends on the activation state of the EV-releasing cell and differs between different cell types [20]. Importantly, the combined presence of different functional molecules in EVs can result in different cellular responses compared to the individual components [21]. Additionally and in contrast to soluble proteins, some membrane-associated proteins may be resistant to inhibition as was shown for neutrophil elastase activity for example [22] or can induce more potent responses as was shown for EV-associated Hsp70 [23]. Taken together, the selective incorporation of cargo determines how and where EVs exert their function.

It is clear that EVs can substantially influence different physiological and pathological processes, including immune responses [24, 25], cancer [26], and cardiovascular diseases [27]. As it is now increasingly recognized that innate immune cells may modulate the adaptive immune response, we will focus in this review on the contribution of EV-mediated cross-talk between cells of the innate and adaptive immune system to the initiation or regulation of immune responses. More specifically, we will focus on macrophages, mast cells, neutrophils, eosinophils, basophils, and NK cells as producers of EVs that modulate T and B cell functions (either by directly targeting these cells or indirectly by targeting DCs). Since DCs are well-known for their role as master regulator of adaptive immunity and because the immune-modulating characteristics of DC-derived EVs, including their use in clinical trials aiming at inducing tumor-specific T cell responses, have been reviewed extensively [25, 28–30], we did not include DC-derived EVs in the current review. Finally, multiple different protocols are used for the isolation of (subsets of) EVs, and EV research is frequently complicated by the possible presence of contaminants in EV samples, e.g., protein aggregates and lipoproteins. Efforts have been made to standardize EV-related experimental methods and documentation [31, 32], and to improve the interpretation of the reviewed data, we concisely indicated the EV isolation methods (i.e., pelleting speed, possibly followed by density gradient ultracentrifugation (DGC)) that were used to obtain the described EV content or their immune modulatory effects.

## Mast cells

Mast cells are tissue-resident cells that are particularly found at sites that are in contact with external environments such as the skin, gut, and airways. Moreover, mast cells can be found in lymphoid tissues, where their presence can significantly increase during inflammatory responses [33–36]. Physiologically, mast cells appear to have a prominent role in orchestrating innate and adaptive immunity required for host defense against microbial infections (parasites, bacteria, virus) and animal venoms. Moreover, these cells are associated both with the induction of immune tolerance but also with the initiation and progression of multiple immune disorders [33, 37, 38]. Although direct cellular contacts and soluble mediator release are two important mechanisms employed by mast cells to exert these functions [2, 11, 39], mast cells release EVs under resting and activated conditions that are indicated to play a role.

Several studies have demonstrated that mast cells can express MHC class II (MHC-II) and co-stimulatory molecules under specific inflammatory conditions (LPS, IFN-γ, IL-4) [40–44], or acquire MHC-II after contact with DCs [45], and thereby regulate effector memory CD4^+^ T cell responses [42–45]. In line with these data, MHC-II was shown to be present on EVs (70,000 g (70K) pellets) from murine bone marrow-derived mast cells (BMMCs) and RBL-2H3 cells (rat basophilic leukemia used as a mast cell model) using electron microscopy in combination with immunolabeling, western blotting, and ELISA [46–48]. In BMMC cell cultures, MHC-II-containing EVs were released both by degranulating [46] and unstimulated BMMCs [49], two conditions that result in the release of two completely different EV subsets based on size, and protein and lipid composition [50]. In an artificial model using hemagglutinin peptide-loaded HLA-DR1-expressing RBL-2H3 cells, EVs from degranulated cells (70K pellets) could significantly induce the proliferation of hemagglutinin-specific Jurkat T cells, but only in the presence of immature DCs [47]. Merely weak T cell activation was observed in the absence of DCs, indicating that mast cell-derived EVs must be taken up by professional antigen-presenting cells or that additional DC-derived costimulatory signals were required for T cell activation. A comparable requirement was shown for MHC-II-positive DC-derived EVs in order to induce substantial effects on T cell responses [25, 51]. So far, no additional data exist that further substantiate a role for mast cell EVs in the transfer of MHC-II-peptide complexes and in direct antigen presentation to T cells. Others demonstrated that antigen (ovalbumin)-primed unstimulated BMMCs can transfer endocytosed and partially processed antigens via EVs (70K pellets) to immature bone marrow-derived DCs (BMDCs) in vitro or to DCs in vivo after EV injection. Interestingly, this EV-associated ovalbumin was far more efficiently presented by DCs than soluble ovalbumin in the presence of LPS. The authors further showed that antigen loading into EVs was dependent on heat shock proteins (Hsp60 and Hsc70), and that the low-density lipoprotein receptor-related protein 1 (LRP1/CD91) expressed on DCs was involved in the uptake of Hsp-positive EVs [52]. Moreover, these mast cell-derived EVs were shown to induce BMDC maturation (upregulated expression of MHC-II, CD80, CD86, and CD40) and IL-12p70 release by DCs. This effect on DCs was likely responsible for additionally observed effects: enhanced B and T cell proliferation both in vitro and in in vivo murine models upon intraperitoneal injection of antigen-primed unstimulated BMMC-derived EVs (increased IL-2 and IFN-γ release, no IL-4). Furthermore, efficient antigen-specific antibody responses (IgG1/IgG2a) could be elicited in mice after subcutaneous injection of these EVs in the absence of conventional adjuvants [48, 52].

Although clear immunomodulatory functions are described for different heat shock proteins [53, 54], their functional role in mast cell EVs other than loading of antigenic cargo has not been demonstrated. It should be noted that although we also identified Hsp60, Hsp70, and Hsp90 in murine spleen-derived and peritoneal mast cell EVs, we did not observe any effects on the maturation of BMDCs upon incubation with EVs isolated from unstimulated mast cells (100K pellets, purified by DGC, or only using 100K pellets comparable to the above described papers) ([50] and our own unpublished data). These contradicting findings indicate that either mast cells derived from different precursors release functionally different EVs, and/or that this difference in functionality is due to different culture conditions employed, such as the required treatment with IL-4 to generate EVs with DC-activating properties [48, 52]. As such, it is likely that mast cells only release immune stimulatory EVs when exposed to specific inflammatory conditions, since it would be physiologically unfavorable when constitutively released EVs continuously activate surrounding DCs and induce B and T cell activation.

Another mechanism of antigen transfer from mast cells to DCs may involve antigen-IgE complexes bound to mast cell EVs. The presence of the high-affinity IgE receptor (FcεRI) on EVs from both unstimulated and degranulated RBL-2H3 cells (EV isolation protocol unknown) and unstimulated BMMCs (120K pellets) was shown [55, 56], though we only detected the Fc receptor γ-chain in EVs isolated (100K pellets purified by density gradient ultracentrifugation) from degranulated murine peritoneal mast cells [50]. Such FcεRI-IgE complexes were shown to be able to transfer antigen from BMMCs to BMDCs in a direct cell contact-dependent manner following induction of mast cell degranulation, although a contribution of EVs was not investigated [57].

Mast cell EVs have also been shown to contain lipases and lipid mediators that may be involved in the modulation of adaptive immune responses. Using a human mast cell line (LAD2), Cheung et al. showed that EVs derived from IFN-α-activated mast cells contain active cytosolic *phospholipase A2* (PLA2G4D), which can be transferred to CD1a-expressing cells (either a CD1a-expressing leukemic cell line or monocyte-derived DCs), leading to the generation and presentation of neolipid antigens. This subsequently induced the activation of lipid-specific CD1a-reactive T cells of psoriasis patients leading to the production of IL-22 and IL-17A [58]. Although EVs were isolated using a “exosome extraction reagent” which is not a generally accepted EV isolation method, PLA_2_ was also shown in EVs isolated after ionomycin-mediated RBL-2H3 activation (100K pellets) [59], and also we found PLA_2_ activity in EVs isolated from degranulated peritoneal mast cells (100K pellets purified by DGC, own unpublished data). Interestingly, PLA_2_ can generate lysophospholipids, such as lysophosphatidylcholine (LPC), which could lead to membrane damage and IL-33 release. In turn, IL-33 can directly signal through ST2 on T cells to induce Th2 cell responses, as was shown in mice [60]. LPC themselves can also directly influence T cell chemotaxis and function [61, 62], NKT cell activation [63], and DC maturation [64]. Furthermore, PLA_2_ can directly induce DC maturation [65]. Besides PLA_2_, RBL-2H3-derived EVs (100K pellets) also contained PLD_2_ activity, arachidonic acid, and its derivatives including prostaglandin E2 (PGE2) and PGD2 [59, 66]. These prostaglandins may directly modulate DCs and T and B cells leading to both pro- and anti-inflammatory effects [67]. Moreover, PLD_2_ hydrolyzes PC to generate choline and phosphatidic acid (PA), which in turn can by hydrolyzed by PLA_2_ into lyso-PA that can inhibit DC function, and effect DC migration, cytokine release (inhibition of TNF-α and IL-12, enhanced IL-10 release), and their capacity to induce Th1 cell differentiation [68, 69]. Together, these findings implicate that mast cell-derived EVs may directly or indirectly be involved in the generation of lipid mediators that can modulate adaptive immune responses.

Finally, it was shown that EVs from degranulated murine peritoneal mast cells (100K pellets purified by density gradient ultracentrifugation) contain biologically active mast cell-specific proteases (carboxypeptidase A3, tryptase, chymase) [50]. Association of proteolytic enzymes to EVs may be a mechanism to effectively position combinations of enzymes at distant sites, where mast cell proteases could influence adaptive immunity by proteolytic processing or degradation of cytokines and chemokines, including the IL-1 cytokine family members IL-18 and IL-33, as well as IL-15 [70, 71]. Moreover, proteases may directly target T cells. In a murine model, it was found that mast cell protease-6 was involved in suppression of Th2 cytokines by activating PAR2 on Th2 cells [72].

Evidence for the direct targeting of T and B cells by mast cell EVs is still limited. Using EV-bead flow cytometry, it was suggested that unstimulated BMMCs release CD40L^+^ EVs (ExoQuick isolation) that were undetectable in culture supernatants upon co-culture with murine splenic B cells suggesting that they were captured by B cells. This transfer of EVs was suggested to be involved in the generation of IL-10 competent B cells [73]. CD40L was also found on BMMC-derived EVs previously [48] and may possibly also influence isotype switching, somatic hypermutation, and plasma and memory B cell formation, as well as DC maturation [74]. Lastly, another group showed that OX40L^+^ EVs derived from unstimulated BMMCs (120K pellets) promote Th2 cell differentiation by targeting anti-CD3/CD28-stimulated T cells in the presence of IL-4 [75]. However, this latter study has several methodological shortcomings, limiting the value of this finding.

## Neutrophils

Neutrophils are circulating cells that are generally rapidly recruited to peripheral sites upon initiation of inflammation, where their main known function is the phagocytosis and killing of invading pathogens and clearance of debris [76]. Moreover, it is increasingly recognized that different neutrophil phenotypes with distinct functions exist [77, 78], that neutrophils also infiltrate secondary lymphoid organs [79], and that neutrophils are more than just final effector cells. Neutrophils are now known to be involved in orchestrating adaptive immune responses by influencing various cell types, either by releasing multiple soluble mediators or via direct cellular contacts. For instance, neutrophils can modulate DC migration, maturation, and their CD4^+^ T cell polarizing capacity [2, 80]. In addition, they can suppress or enhance B cell, CD4^+^ T cell, and γδ T cell functions (independent of MHC), or even act as antigen-presenting cells under specific activating conditions and thereby drive antigen-specific T cell responses [10, 79]. Limited studies have been performed so far indicating that neutrophil-derived EVs may regulate some of these immune modulatory processes. Nevertheless, already quite some research has been conducted on the characterization of vesicles released from resting or differentially activated neutrophils, which revealed the presence of several molecules that have the capacity to modulate adaptive immunity.

Human neutrophil degranulation induced by *N*-formyl-methionyl-leucyl-phenylalanine (fMLP) resulted in the release of EVs (160K pellets) that have inhibitory effects on monocyte-derived DCs [81]. These EVs altered the morphology of LPS-activated DCs by inhibiting the formation of dendrites and reduced the phagocytic activity and maturation (lower expression of HLA-DP/DQ/DR, CD40, CD80, CD83, CD86), resulting in an attenuated capacity to induce T cell proliferation. Furthermore, the release of the cytokines IL-8, IL-10, IL-12, and TNF-α by LPS-activated DCs was inhibited by these EVs, while the release of TGF-β1 was increased [81]. These effects on cytokine release were in line with previous observations showing that resting, and LPS- or zymosan A-activated macrophages decrease the release of pro-inflammatory cytokines (TNF-α, IL-6, IL-8) and increase TGF-β1 release upon targeting by human neutrophil-derived EVs (160K pellets, or concentrated culture supernatants) [82–84]. Phosphatidylserine (PS) is one of the modulatory molecules present in the membranes of neutrophil-derived EVs [81, 85, 86] and was shown to be involved in mediating anti-inflammatory effects in line with the generally known immunosuppressive function of this phospholipid [87]. Indeed, blocking PS with Annexin V before adding the EVs to the activated DCs significantly reduced the above described effects [81]. However, a follow-up study from the same group showed that the presence of PS on the EV surface is required for the binding and/or uptake of EVs, but not sufficient to induce the release of TGF-β1 from macrophages [84]. Other EV-associated factors responsible for the release of TGF-β1 were not further indicated.

The increased release of TGF-β1 by DCs and macrophages upon incubation with neutrophil-derived EVs can have diverse effects on the adaptive immune system, but is primarily known for its suppressive effects on CD4^+^ Th1 and Th2 cell differentiation, and induction of regulatory T cells. Moreover, it can promote Th9 and Th17 cell generation, limit CD8^+^ T cell and B cell functions, influence DC migration, and induce anti-inflammatory pathways in DCs leading to dampening of T cell responses [88]. These findings suggest that neutrophil-derived EVs have an indirect, primarily anti-inflammatory effect on the adaptive immune system by influencing mediator release by targeted DCs and/or macrophages. However, another study showed direct effects of EVs (100K pellets) derived from UV-B light-induced apoptotic human neutrophils on CD4^+^ T cells [89]. These EVs were found to bind anti-CD3/CD28-activated CD25^−^CD127^+^ (naïve and resting central memory) and CD25^+^CD127^+^ (recently activated effector) T cells and reduced their release of TNF-α. In contrast, these EVs only suppressed the proliferation of CD25^−^CD127^+^ cells, likely by reducing both IL-2 secretion and CD25 upregulation upon stimulation [89]. The neutrophil granule proteins MPO and arginase-1 were detected in the EV preparations, and although their described immunosuppressive properties (described below), they were not found to mediate the suppressive effects on T cells [89]. In addition, EVs from neutrophils are enriched in CD11b (possibly enhanced in EVs from apoptotic neutrophils) [85, 86, 89, 90], which may be involved in their binding to activated T cells [91]. Collectively, these data support the immune-suppressing roles of neutrophil-derived EVs mediated by DCs and macrophages, and indicate that EVs released by apoptotic neutrophils may contribute to the prevention of uncontrolled activation and expansion of resting T cells, thereby supporting the termination of immune reactions.

Besides these studies that explored immune-modulating functions of neutrophil-derived EVs, others have focused on the characterization of their release, size, and content, and identified several EV-associated molecules that can potentially influence DC and T cell functions. In this regard, it was shown that Annexin A1 is present on the surface of EVs released from fMLP-stimulated neutrophils (100K pellets) [92–94]. Annexin A1 is an immune-modulating mediator with a broad range of actions including immune-suppressive (inhibition of T cell proliferation and Th2 cell development, induction of regulatory T cells) and activating (favoring Th1- and Th17-mediated responses) capacities [95]. Arginase-1 and lactoferrin are other anti-inflammatory proteins identified in EVs derived from apoptotic neutrophils as described above [89], and EVs (15.7K pellets) from resting or *Staphylococcus aureus*-activated neutrophils, respectively [90]. Arginase-1 depletes the nonessential amino acid L-arginine which can result in limited T cell proliferation and impaired T cell function [10]. Lactoferrin can increase DC recruitment to sites of infection and was also shown to inhibit DC migration to draining lymph nodes. Moreover, contradicting results were found on the role of lactoferrin in the induction of DC maturation [2, 76]. Furthermore, several proteases have been identified in neutrophil-derived EVs (15.7–200K pellets) that may influence adaptive immunity, such as myeloperoxidase (MPO), cathepsin G, proteinase 3, and elastase [85, 90, 93, 96, 97]. Interestingly, EV-associated MPO was found more effective in influencing epithelial cell function when compared to recombinant soluble MPO, possibly due to targeting MPO activity or an increased MPO stability [97]. Although these proteases are also found in the so-called neutrophil extracellular traps (NETs), which potentially contaminate pelleted EV fractions, we confirmed the association of elastase to EVs (10K and 100K pellets) after their purification by DGC (own unpublished data). Anti-inflammatory effects have been described for MPO, which can suppress various aspects of DC function in vitro and in vivo, including their migration, antigen uptake, maturation, and subsequent induction of T cell responses [98]. Elastase, cathepsin G, and proteinase 3 have the capacity to inactivate several cytokines (including IL-2, IL-6, TNF-a, IL-15, and IL-33) and to shed IL-2 and IL-6 receptors from the plasma membrane by proteolytic cleavage, and may thereby inhibit diverse T cell functions [71, 99, 100]. Also, elastase has been described to induce TGF-β secretion by DCs and to decrease their capacity to induce T cell proliferation [101], which may in part explain the above described TGF-β-inducing effects in DCs and macrophages of neutrophil EVs.

Next to these immune-suppressive functions, it was found that elastase, cathepsin G, and proteinase 3 can activate IL-36 which can induce DC maturation, enhance Th1 or Th17 cell induction, and inhibit regulatory T cell development [102]. Moreover, elastase is able to potentiate the activation of γδ T cells via the activation of protease-activated receptor 1 (PAR1) [103] and to critically influence the generation of human Th17 cells from naive CD4^+^ T cells by cleaving IL-8 into a truncated form, which is subsequently required for Th17 cell development [80]. Another molecule that was identified in EVs (100K pellets) released from fMLP-activated human neutrophils was LTB_4_ (together with LTB_4_-synthesizing enzymes), which was shown to stimulate the directional migration of neutrophils themselves [93, 104]. Although possible consequences of EV-associated leukotriene were not further investigated on other cells, it may also enhance DC migration to sites of infection and/or the draining lymph nodes [105] and act as a chemoattractant for CD4^+^ and CD8^+^ T cells [106].

## Macrophages

Macrophages are important phagocytic cells distributed in essentially all tissues, and are implicated in multiple different responses that are essential for host defense against invading pathogens, tissue development, and homeostasis (including tissue integrity and healing). Upon infection, macrophages can rapidly recruit various innate and adaptive immune cells. In addition, they can be involved in mediating CD4^+^ and CD8^+^ T cell responses and in the activation of B cells and invariant NKT cells [107–109]. Although cytokines play a prominent role in these effects, there is increasing evidence indicating that macrophage-derived EVs also contribute to this control of adaptive immunity.

Several groups demonstrated that macrophage-derived EVs directly or indirectly modulate DC and T cell responses. For example, mycobacterial-infected murine macrophages (J774 cell line) were found to release EVs (100K pellets, followed by DGC purification) that contain mycobacterial antigens and have the ability to induce BMDC maturation (increased expression of CD83, CD86, and MHC-II) and IL-12p40 expression [110]. Moreover, these EVs induced the proliferation and activation of CD4^+^ and CD8^+^ T cells in vitro and in vivo after intranasal injection in mice, which resulted in the induction of a population of effector memory T cells. The presence of DCs was required for the optimal induction of T cell responses [110], comparable to the above described actions of mast cell- and DC-derived EVs. In contrast, another study showed that MHC-II^+^/CD86^+^ EVs (10K pellets) derived from ATP-activated mycobacterial-infected bone marrow-derived macrophages (BMDM) can efficiently present endogenously processed antigens to BB7 T hybridoma cells and P25 TCR-transgenic naive T cells in vitro without need for additional DCs [111]. However, this seeming discrepancy can be the result of different EV populations studied due to different isolation protocols used or to the different requirements for co-stimulation in the read-out T cell models.

Two other studies showed that DCs and macrophages themselves are major target cells of macrophage-derived EVs in mice. After intranasal injection of EVs (100K pellets, followed by DGC purification) derived from mycobacteria-infected human THP-1 cells, targeting of both DCs and macrophages in the lungs was demonstrated [112], while after subcutaneous injection of EVs (100K pellets, no differential centrifugation) derived from a murine macrophage cell line (RAW264.7), targeting of DCs and macrophages was demonstrated in the draining lymph nodes [113, 114]. This latter study showed that EVs derived from macrophages with an M1 phenotype induced the release of Th1 cell-promoting cytokines (IL-12, INF-γ) by both RAW264.7 macrophages and a DC cell line JAWSII, and induced a stronger antigen-specific cytotoxic T cell response in vivo when administered together with a peptide vaccine. Instead, EVs released by M2-like macrophages enhanced IL-4 and IL-10 release by macrophages and DCs [114]. Finally, EVs (100K pellets, followed by DGC purification) derived from mycobacterial-infected murine macrophages (RAW264.7 cells) induced the release of multiple cytokines and chemokines (e.g., TNF-α, CCL3, and CCL5) from uninfected macrophages that significantly enhanced the transmigration of TCR-β^+^ T cells in in vitro assays, and may also influence the migration of DCs [112].

Besides the analysis of functional consequences of macrophage-derived EVs on DCs or T cells, several studies characterized EV content and/or their effects on uninfected macrophages which revealed immune modulatory components. As indicated, macrophage-derived EVs can contain microbial components and may as such transfer both antigens and activating signals to DCs, thereby promoting the development of adaptive immune responses comparable to what was described above for mast cell EVs. Glycopeptidolipids (GPLs; a major cell wall constituent of *Mycobacterium avium*), lipoarabinomannan (LAM), and 19-kDa lipoprotein were found in EVs (100K pellets, followed by DGC purification) derived from mycobacteria-infected macrophages (murine J774 cells and human THP-1 cells) and were transferred to and activated uninfected macrophages in a TLR-dependent manner [113, 115]. Moreover, intranasal injection of these EVs in mice induced significant IL-12p40 and TNF-α release in the lung 1 day after injection, which may support the Th1 cell-inducing effects of M1 macrophage EVs described above. Furthermore, LPS was found in EVs from *Salmonella*-infected THP-1 cells, which induced TLR-4-dependent BMDM activation [113]. Also GP63, a surface protease of the *Leishmania mexicana* parasite, was shown to be present on EVs (100K pellets, followed by DGC purification) derived from infected J774 macrophages, yet functional implications were not further investigated [116].

It is yet poorly understood which components present in macrophage EVs other than microbial molecules can regulate the modulatory effects on DC and T cell responses. Yet, several molecules have been identified that are potentially involved. Increased amounts of Hsp70 for example were shown to be present in EVs (100K pellets, followed by DGC purification) derived from mycobacteria-infected RAW264.7 cell macrophages, which may contribute to the pro-inflammatory properties of these EVs [117]. However, Hsp70 is also associated with a tolerance-promoting potential [54]. The ultimate actions of Hsp70 may depend on additional EV-associated signals, the expression of Hsp70 (membrane-exposed or luminal), or the exact form of Hsp70 present in EVs (e.g., protein modifications). Interestingly for IL-1β, it was shown that it is present in its unprocessed and mature, bioactive form in EVs (isolated by Annexin bead pulldown) derived from ATP-activated human THP-1 cells [118] and murine BMDM-derived EVs (100K pellets) upon induction of inflammasome signaling upon activation with LPS/nigericin (a potent activator of the NRLP3 inflammasome) [119]. IL-1β can among others be involved in the modulation of DC function and migration, and in the expansion and differentiation T and B cells [120–122]. However, the presence of IL-1β in EVs was not supported by Qu et al., using EVs derived from murine BMDMs [123]. Also, TNF-α and several chemokines (e.g., CCL2, CCL3, CCL4, and CCL5) were found in EVs (100K pellets) derived from LPS/nigericin-activated BMDMs and LPS-stimulated RAW264.7 cells [119, 124]. Although the mechanisms by which these EV-associated molecules can exactly function are still unclear, they may be involved in the enhanced activation and/or migration of DCs and T cells. Furthermore, the EVs from LPS-stimulated RAW264.7 cells contained miRNAs that can potentially target proteins involved in TLR and chemokine signaling, and as such may have the capacity to modulate DC-driven responses [124]. Another group showed that EVs (70K pellets) released from macrophages (P388D1 cell line) can contain complement C3 fragments on the outer membranes, which may be involved in enhancing T cell proliferation in the presence of antigen-presenting cells [125]. Also proteins of the leukotriene pathway have been identified in EVs (100K pellets) released by human monocyte-derived macrophages, and these EVs had the capacity to convert LTA_4_ to LTB_4_ and LTC_4_ [126]. Functional consequences of this EV-mediated leukotriene synthesis were not further addressed, but may encompass enhanced migration, activation, and antigen presentation by DCs, reduced IL-12 release by DC (thus enhanced Th2 cell development), and migration of, and cytokine production by CD4^+^ and CD8^+^ T cells [106]. One other immunomodulatory molecule that was recently demonstrated to be released within EVs (both 10K and 100K pellets) from murine pulmonary macrophages stimulated with bacteria or bacterial components is IL-36γ. The release of these EVs was again further enhanced by the additional activation with ATP [127]. How this encapsulated IL-36γ will be released from EVs and how it might influence adaptive immunity remains elusive, but DCs, B cells, and T cells can express IL-36R, which activation can induce DC maturation, enhance Th1 or Th17 cell generation, and inhibit regulatory T cell development [128].

## Eosinophils, basophils, and NK cells

Eosinophils represent a small number of circulating granulocytes that primarily infiltrate sites of helminth infection or allergic inflammation, where their release of multiple (pre-formed) effector molecules mainly attributes to both physiologic and pathogenic effects, respectively. Moreover, eosinophils are found in several tissues under steady-state conditions where they maintain tissue, metabolic, and immune homeostasis [14]. Eosinophils can modulate DC functions, and T and B cell responses [2, 12], but until now the contribution of eosinophil-derived EVs in these processes is not addressed. One possible mechanism may involve the EV-mediated (100K pellets) transfer of the cationic proteins major basic protein (MBP) and eosinophil peroxidase (EPO). Eosinophils were shown to increase the release of EVs containing these proteins upon IFN-γ stimulation [129, 130]. Both MBP and EPO may enhance DC maturation (upregulated expression of CD80, CD83, and CD86 [131, 132]. Moreover, EPO can favor DC-driven Th2 cell development by increasing CCR7 and OX40L expression and the release of IL-6 and TNF-α by murine BMDCs, while significantly inhibiting IL-12p40 production [3, 132].

Basophils are found at low frequencies in the circulation and are primarily associated with the resolution of parasitic infections and with detrimental roles in allergic diseases, and autoimmune diseases as well [13]. Despite some clear evidence for their ability to influence DC function and DC-driven Th2 cell responses [2], there is currently no data available on basophils either as producers or targets of EVs.

NK cells are granular innate immune cells of the lymphoid lineage that are found in the circulation and in (secondary lymphoid) tissues, where they are primarily involved in the killing of virus-infected cells and tumor cells. NK cells can influence several immune responses and immune-related disorders by releasing several cytokines and chemokines that for example influence DC function or Th1 cell development [7, 16, 133]. So far, there is no clear evidence that NK cell-derived EVs have any modulatory effect on DC, T cell, or B cell functions. However, although several groups described the capacity of NK cell-derived EVs to be involved in tumor cell lysis [134–136], one study also investigated cytotoxic effects on immune cells. Lugini et al. showed that EVs (100K pellets) from both unstimulated and activated NK cells were able to lyse PHA-activated, but not resting PBMCs [134]. The cytotoxic molecules perforin and FasL were detected in NK-derived EVs (100K pellets, with and without DGC purification), and particularly perforin was found to be responsible for the lytic effects [134–136]. Since PHA is a selective T cell mitogen, these findings implicate a selective effect on activated T cells and suggest a possible role for these EVs in dampening T cell-mediated inflammation.

## Concluding remarks

Based on the summarized literature in this review, it is becoming clear that the EVs released by different innate immune cells can significantly influence the course of adaptive immune responses or may at least have the capacity to do so based on their cargo. An overview of the molecules associated with innate immune cell-derived EVs that are potentially involved in these processes is summarized in Table 1. A point of caution is the fact that some of the functions and cargo attributed to EVs is not truly EV-associated but based on co-isolated or aggregated material during the EV isolation process, especially since in many of the referred studies, EVs were isolated by centrifugation only protocols.

**Table 1:** Overview of molecules associated with innate immune cell-derived EVs that were either shown to be involved in the modulation of adaptive immune responses or that are identified in innate immune cell-derived EVs and have potential immune-modulatory capacity

Cell type	EV-associated molecule(s)	(Potential) target cell	(Potential) target molecule	(Potential) effects	References
Mast cells					
EV content shown to be involved in immune modulation	MHC-II	DCs, T cell		T cell activation	[46–49]
	Endocytosed antigens	DCs		Antigen transfer	[52]
	Hsp60, Hsc70	DCs		- Cargo selection and uptake by DCs - Immune activation or suppression	[50, 52]
	PLA_2_	DCs	Phospholipids	- Generation of neolipid antigens and lysophospholipids - DC activation	[58, 59]
	CD40	B cells		EV binding	[48, 73]
EV content with potential immune-modulatory functions	FcεRI	DCs		Transfer of IgE-antigen complexes	[50, 55, 56]
	PLD_2_		Phosphatidyl-choline (PC)	Phosphatidic acid (PA) generation may lead to lyso-PA which inhibits DC function and affects Th1 cell generation	[59, 66]
	PGD2, PGE2	DCs, T cells, B cells		Variable, immune activation or suppression	[59, 66]
	Proteases	T cells	Cytokines	- Cytokine processing and inactivation (e.g., IL-15, IL-18, IL-33) - Th2 cell induction	[50]
Neutrophils					
EV content shown to be involved in immune modulation	Phosphatidyl-serine (PS)	DC		- EV binding/uptake - Induction of TGF-β1 release	[81, 85, 86]
EV content with potential immune-modulatory functions	CD11b	T cell		EV binding to activated T cells	[85, 86, 89, 90]
	Elastase	DCs, T cells	Cytokines, membrane receptors	- Cytokine inactivation (e.g., IL-2, TNF-α), activation (IL-36), and processing (IL-8) - Receptor shedding (CD25, CD126) - Induction of TGF-β secretion by DCs, suppressing DC stimulatory capacity - Th17 cell induction - Activating γδ T cells	[85, 96]
	MPO	DCs		Suppression of migration, antigen uptake, maturation, and cytokine release	[85, 90, 93, 96, 97]
	Proteinase 3		Cytokines, membrane receptors	- Cytokine inactivation (e.g., IL-2, TNF-α) and activation (IL-36) - Receptor shedding (CD25)	[85]
	Cathepsin G		Cytokines, membrane receptors	- Cytokine inactivation (e.g., IL-2, TNF-α, IL15, IL-33) and activation (IL-36) - Receptor shedding (CD25, CD126)	[93]
	LTB_4_	DCs, T cells		Increased migration	[93, 104]
	Annexin A1	DCs, T cells		- Induction of Th1, Th17, or regulatory T cells, limiting Th2 cell development - Suppression of T cell proliferation	[92–94]
	Lactoferrin	DCs		Modulation of migration and maturation	[90]
	Arginase-1		L-Arginine	Suppression of T cell proliferation and function	[89]
Macrophages					
EV content shown to be involved in immune modulation	MHC-II-peptide complexes	DCs, T cells		- MHC-II-peptide complexes transfer to DCs - T cell activation	[110, 111]
EV content with potential immune-modulatory functions	Microbial components	DCs		Transfer of antigens and activating signals	[110, 113, 115, 116]
	Hsp70	DCs		Variable, immune activation or suppression	[117]
	IL-1β	DCs, T cells, B cells		- Increased DC migration and IL-12 release - Increased T cell expansion, inducing IFN-γ- and IL-17-producing CD4^+^ T cells and granzyme B^+^ CD8^+^ T cells - Induction of regulatory B cells	[118, 119]
	TNF-α and CCL 2–5	DCs, T cells		Enhancing activation and/or migration	[119, 124]
	C3 fragments	DCs, B cells		Enhancing T cell proliferation	[125]
	Leukotriene-related proteins		LTA_4_	- Increased migration, activation by DCs, reducing IL-12 release - Increased migration and cytokine production by T cells	[126]
	IL-36γ	DCs, T cells, B cells		- Increased DC maturation - Enhanced Th1 or Th17 cell generation, limiting regulatory T cell development	[127]
Eosinophils					
EV content with potential immune-modulatory functions	MBP	DCs		Enhancing DC maturation	[129, 130]
	EPO	DCs		- Increased maturation, altered cytokine release - Favoring Th2 cell development	[129, 130]
NK cells					
EV content with potential immune-modulatory functions	Perforin and FasL	Activated T cells		Cell lysis	[134]

In general, it can be concluded that the majority of the effects induced by EVs derived from innate immune cells are mediated via targeting of DCs and subsequent antigen delivery or influencing DC maturation or migration. However, some papers described also direct effects on T cells by innate immune-derived EVs. Moreover, while mast cell- and macrophage-derived EVs appeared to be primarily pro-inflammatory, EVs released from either activated or apoptotic neutrophils are thus far mainly associated with immune-suppressive functions. Nevertheless, since the nature of the activating stimulus can significantly affect the content and biological functions of released EVs, their capacity to either enhance or inhibit adaptive immunity may significantly alter when isolated after culturing innate immune cells under suppressive or inflammatory conditions that are thus far unexplored. Due to lack of research, immune-modulatory functions of eosinophil, NK cell, and basophil EVs still remain largely elusive. Besides, it is striking that evidence for a possible role of T cell- and B cell-derived EVs in influencing innate immune cell functions is understudied, with the exception of two studies from one group describing a role of anti-CD3/CD28-activated CD4^+^ T cell EVs (20K or 100K pellets, unknown) in inducing mast cell degranulation and cytokine release [137]. Moreover, it was demonstrated that T cell-derived EVs (20K pellets) elicited mast cell activation resulting in the upregulation of several clusters of genes, especially cytokine- and chemokine-related ones, including IL-24 [138]. Based on these findings, it is well possible that T cell EVs also target other innate immune cells, especially at inflammatory sites were activated T cells and multiple different innate immune cell types are present simultaneously.

In conclusion, multiple studies performed to date indicate a significant role for EVs in the communication between innate and adaptive immunity, and especially the contribution of mast cell-, neutrophil-, and macrophage-derived EVs to this cross-talk is investigated thus far. A good understanding of the capacity of EVs released by innate and adaptive immune cells to control the two arms of the immune system may reveal new insights on the complex regulatory circuits operating in the immune system. Moreover, this can uncover new possibilities for the use of EVs (or their components) in controlling immune responses, either as a therapy or as a target in future immune-modulating treatments.
